# Oral Administration of Escin Inhibits Acute Inflammation and Reduces Intestinal Mucosal Injury in Animal Models

**DOI:** 10.1155/2015/503617

**Published:** 2015-06-25

**Authors:** Minmin Li, Chengwen Lu, Leiming Zhang, Jianqiao Zhang, Yuan Du, Sijin Duan, Tian Wang, Fenghua Fu

**Affiliations:** Key Laboratory of Molecular Pharmacology and Drug Evaluation (Ministry of Education of China), School of Pharmacy, Yantai University, Yantai, Shandong 264005, China

## Abstract

The present study aimed to investigate the effects of oral administration of escin on acute inflammation and intestinal mucosal injury in animal models. The effects of escin on carrageenan-induced paw edema in a rat model of acute inflammation, cecal ligation and puncture (CLP) induced intestinal mucosal injury in a mouse model, were observed. It was shown that oral administration of escin inhibits carrageenan-induced paw edema and decreases the production of prostaglandin E2 (PGE2) and cyclooxygenase- (COX-) 2. In CLP model, low dose of escin ameliorates endotoxin induced liver injury and intestinal mucosal injury and increases the expression of tight junction protein claudin-5 in mice. These findings suggest that escin effectively inhibits acute inflammation and reduces intestinal mucosal injury in animal models.

## 1. Introduction

It is well known that glucocorticoids (GCs) and nonsteroidal anti-inflammatory drugs (NSAIDs) are the most common medications for acute and chronic inflammation, such as sepsis, rheumatoid arthritis, and ulcerative colitis [1, 2]. However, GCs have multiple side effects such as inhibiting the immune system which is also associated with a risk of osteoporosis and an increased susceptibility to slowing wound healing [3, 4]. NSAIDs have been associated with high risk of gastrointestinal adverse reaction [5].

**Figure 1:**
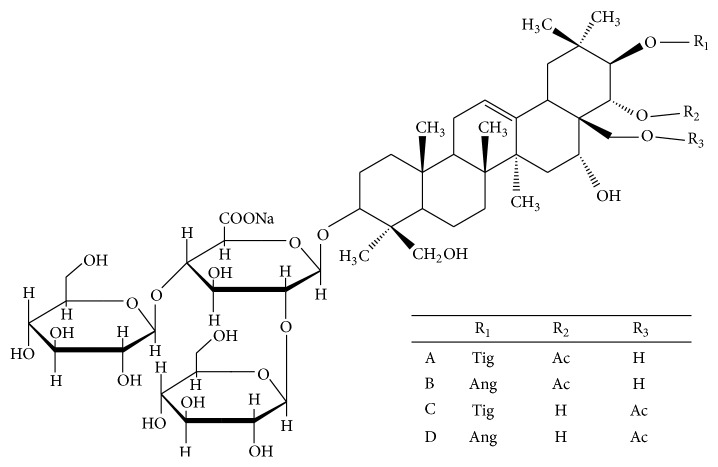
The structure of escin.

Escin is a natural mixture of triterpene saponins extracted from the seeds of* Aesculus chinensis* Bge. or* Aesculus wilsonii* Rehd., which mainly consist of A, B, C, and D escin (Figure 1). Accumulating experimental evidence in previous studies suggests that intravenous administration of escin exerts potent anti-inflammatory and antiedematous effects [6–9]. It can inhibit acetic acid induced vascular permeability in an acute inflammation mouse model and granuloma formation in a subchronic inflammatory rat model. At present, escin injection has been widely used clinically to prevent inflammatory edema after trauma such as fracture and operation in China [10, 11].

However, escin injection can cause some adverse reactions involving multiple organs and systems; the most common reactions are phlebitis and allergic reaction [12]. Phlebitis occurring at the first day after intravenous medication accounts for 70% and can cause physical and psychological pain, which directly affects its clinical use. The adverse reactions induced by escin injection are probably associated with the usage, pH value, diluted solution, impurity, and so forth [13].

Now there is no good way to solve this problem, although some reports showed that administration of certain medications could alleviate the adverse reaction [14]. Oral administration of escin could prevent phlebitis or other adverse reactions which are caused by intravenous injection. So we choose carrageenan-induced paw edema model to examine whether oral administration of escin also has similar anti-inflammatory effects, which can provide the experimental basis for the clinical application of escin tablet.

Sepsis is characterized by a severe inflammatory response to infection. It remains a major cause of morbidity and mortality in critically ill patients. It has been recognized that intestinal epithelial barrier dysfunction and increased intestinal permeability after thermal injury, trauma, severe infection, and so forth may be the inciting incident that ultimately leads to sepsis [15]. Because the gastrointestinal tract is known to be a large pool of bacteria and endotoxin, the intestinal epithelial barrier appears to play a critical role in preventing the translocation of luminal bacteria and endotoxin into systemic organs and tissues via lymphatic channel and bloodstream. So how to protect and reduce the damage of the intestinal mucosa and how to promote the reconstruction of the intestinal mucosa have been the important topics of research in sepsis for many years. However, even though sepsis is timely recognized, there is no effective therapy to sepsis, except antibiotics, fluids, and vasopressors [16].

Previous studies suggest that oral administration of escin can protect against gastric ulcer and promote gastrointestinal transit in animal models [17, 18]. However, whether escin has the protective effects on intestinal epithelial barrier dysfunction is unknown. So in this study, we investigated the effects of escin after oral administration on intestinal mucosal injury in cecal ligation and puncture (CLP) induced mouse model of sepsis.

## 2. Material and Methods

### 2.1. Animals and Drugs

Male Wistar rats (weight, 160–200 g) and Swiss mice were purchased from the Experimental Animal Center of Shandong Luye Pharmaceutical Co., Ltd. (Yantai, China). The animals were kept in air conditioned rooms (temperature, 23 ± 2°C) on a 12 h light-dark cycle, with free access to food and water. Animal experimental procedures were carried out in accordance with the National Institutes of Health regulations on the use and care of animals for scientific purpose and approved by the Ethics Committee of Yantai University.

Escin for oral administration (sodium aescinate tablets, batch number: 20130214) and escin for injection (sodium aescinate for injection, batch number: 1313084) were both obtained from Shandong Luye Pharmaceutical Company Limited (Yantai, China). The dexamethasone (Dex) sodium phosphate injections were purchased from the Cisen Pharmaceutical Company (Jining, China; batch number: 120229203).

### 2.2. Carrageenan-Induced Paw Edema Model

Rats were randomly divided into the following groups with eight rats in each: vehicle group, Dex group (6 mg/kg, i.v.), escin for injection group (1.8 mg/kg, i.v.), and escin for oral administration groups (5, 10, and 20 mg/kg, p.o.). All groups received respective medications 1 h before the carrageenan administration. Paw edema was induced by subcutaneous injection of 0.1 mL of 1% carrageenan solution in saline into subplantar region of the right-hind paw of each rat. Paw volume was measured before the irritant injection and at selected intervals (1, 2, 3, 4, 5, 6, 8, 12, 18, and 24 h) thereafter with a plethysmometer (YLS-7A, Shandong Academy of Medical Sciences, China). For each animal, results were expressed according to the increase in paw volume (mL) calculated by subtracting the basal volume.

### 2.3. Histopathological Examination of Paw Edema Tissues

Four hours after carrageenan injection, the rats were anesthetized with choral hydrate (10%); tissues of the right-hind paw were cut and immersed into 4% formaldehyde for 24 h. Then the tissues were embedded in paraffin, sectioned (5 *μ*m) using a microtome, and stained with hematoxylin and eosin (H-E). The damage was determined by microscope analysis (magnification, 400x; Olympus BX41, Japan).

### 2.4. ELISA for PGE2 and COX-2 Assay

Four hours after carrageenan injection, the rats were anesthetized with choral hydrate; tissues of the right-hind paw were separated on ice and homogenized with ice-cold saline for a 10% (w/v) homogenate. The level of PGE2 (Shanghai Xitang Biological Technology Co., Ltd., Shanghai, China; catalog number: F16550) and COX-2 (Shanghai Xitang Biological Technology Co., Ltd., Shanghai, China; catalog number: F15265) in the paw was determined using ELISA kit.

### 2.5. Cecal Ligation and Puncture (CLP) Model

CLP mice were determined according to previous method with minor modifications [19]. Briefly, mice were randomly divided into six groups: sham, CLP, dexamethasone (Dex) (reference drug, 5 mg/kg, p.o.), escin 5 mg/kg (p.o.), escin 10 mg/kg (p.o.), and escin 20 mg/kg (p.o.). Mice were treated with the same volume of either normal saline or drug before CLP. Two hours later, mice were anesthetized by intraperitoneal injection of chloral hydrate (350 mg/kg), the abdomen was sterilized with iodophor, and a ventral midline abdominal incision (1-2 cm) was made. The cecum was exteriorized, ligated with a sterile silk suture 1 cm from the tip, and double punctured with a 20-gauge needle. The cecum was squeezed to extrude a small amount of fecal material and was returned to the abdominal cavity. The incision was sutured and kept clean by iodophor. Sham-operated mice were treated as described above with the exception of the ligation and puncture of the cecum. Six hours after operation, mice were anaesthetized by chloral hydrate. Blood samples were collected and centrifuged; plasma samples were stored at −20°C for analysis of endotoxin and inflammatory mediators. Liver and intestinal tissues were collected for hematoxylin and eosin staining or snap-frozen until later analysis.

### 2.6. Histopathological Examination of Liver and Ileum Pathology

Tissues of liver and ileum near ileocecus were cut and immersed into 4% formaldehyde for 24 h. Then the tissues were embedded in paraffin, sectioned (5 *μ*m) using a microtome, and stained with hematoxylin and eosin (H-E). The damage was determined by microscope analysis (magnification, 400x; Olympus BX41, Japan).

Damage of the mucosa was observed as follows: Grade 0: there is normal structure of the intestinal mucosa; Grade 1: the subepithelial gap broadened at the apex of the villus, which was accompanied by capillary blood congestion; Grade 2: the subepithelial gap further broadened and epithelial layers separated from the lamina propria; Grade 3: a lot of epithelial layers separated from both sides of villi and a few villi exfoliated; Grade 4: lamina propria was exposed because lots of villi exfoliated; Grade 5: the lamina propria digested and disintegrated, leading to ulceration.

### 2.7. Measurement of Endotoxin and Diamine Oxidase (DAO) Activity in Plasma by ELISA

Plasma was collected for endotoxin analysis, according to the instruction of the ELISA kit (Zhanjiang Bokang Marine Biological Co., Ltd., China). DAO activity in plasma was determined using a commercially available ELISA kit (Shanghai Xinyu Biological Co., Ltd., China).

### 2.8. Western Blot for Claudin-5

Tissues of ileum near ileocecus were separated and homogenized on ice in cold lysis buffer (Beyotime, China) plus 1 : 100 volume of phenylmethylsulfonyl fluoride (PMSF). The homogenate was centrifuged at 14,000 ×g for 5 min at 4°C. The supernatant was aliquoted and protein concentrations were determined using a bicinchoninic acid (BCA) protein assay kit (Beyotime, China). The samples were thawed on ice and mixed with 4x sample buffer (Invitrogen, Carlsbad, CA) and were heated at 100°C for 5 min. Equivalent amounts of proteins (40 *μ*g) were loaded on 10% Tris-glycine, SDS-polyacrylamide gels for fractionation at 90 V. PageRuler Prestained Protein Ladder (Thermo Scientific, Lithuania) was used as markers. Protein on the gel was blotted onto nitrocellulose membranes at 200 mA for 60 min at 4°C. After transfer, the membranes were incubated with blocking buffer (5% skim milk in wash buffer) for 2 h at room temperature and washed three times (5 min/wash) with 0.1% Tween 20 in Tris-buffered saline (TBST). Incubation with antibody (Claudin-5, Abcam Company, catalog: ab53765), in diluent buffer (5% bovine serum albumin and 0.1% Tween 20 in TBS), was performed overnight at 4°C (1 : 1000 dilution). Membrane was then washed three times (5 min each) with TBST. The primary antibody was probed with horseradish peroxidase-conjugated IgG secondary antibody (Beyotime, China; 1 : 5000 dilution) for 2 h, washed three times in TBST, and processed with enhanced chemiluminescence (ECL) detection reagents from Amersham Pharmacia Biotechnology (Piscataway, NJ). The processed membrane was then exposed to photographic films for visualization of signal. The beta-actin western blot was performed for each membrane as an internal control of protein loading.

### 2.9. Statistical Analysis

The data are expressed as means ± SD. Data was analyzed using one-way ANOVA with the Bonferroni post hoc test for multiple *t*-tests. A value of *P* < 0.05 was accepted as indicating a statistically significant difference among groups.

## 3. Results

### 3.1. Effects of Escin after Oral Administration on Carrageenan-Induced Paw Edema

**Figure 2:**
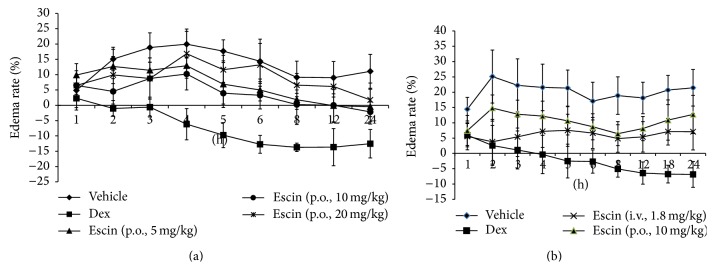
Effects of escin after oral administration on carrageenan-induced paw edema. (a) Rats were assigned to five groups: vehicle group, Dex group, and escin for oral administration groups at doses of 5, 10, and 20 mg/kg. (b) Rats were assigned to four groups: vehicle group, Dex group, escin for injection group (i.v., 1.8 mg/kg), and escin for oral administration group (p.o., 10 mg/kg). *n* = 8. A value of *P* < 0.05 was accepted as indicating a statistically significant difference among groups.

Compared with vehicle group, Dex (6 mg/kg) significantly reduced carrageenan-induced paw edema in rats at 2, 3, 4, 5, 6, 8, 12, and 24 h timing points (*P* < 0.01). 5 and 10 mg/kg, but not 20 mg/kg, escin significantly inhibited the development of paw edema at 3, 4, 5, 6, 8, 12, and 24 h timing points (*P* < 0.05 or *P* < 0.01). Injection (1.8 mg/kg) or oral administration (10 mg/kg) of escin did not show significant differences on inhibiting paw edema at all timing points except the 2 and 3 h timing points (Figure 2).

### 3.2. Effects of Escin after Oral Administration on Histological Pathology of the Edema Paws

**Figure 3:**
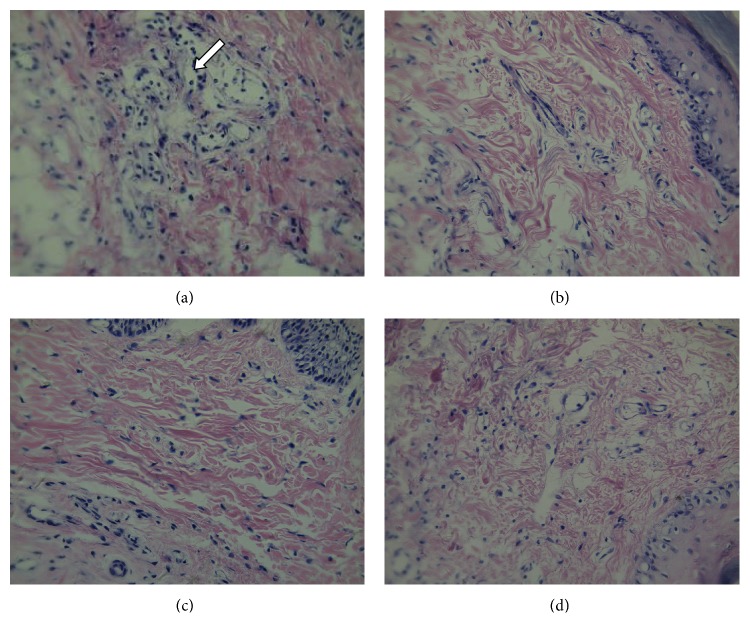
Effects of escin after oral administration on histological pathology of the edema paws in rats. Tissues of the right-hind paw were stained with hematoxylin and eosin (H-E). (a) Vehicle group; (b) Dex group; (c) escin (i.v., 1.8 mg/kg) group; (d) escin (p.o., 10 mg/kg) group. Arrows indicate the inflammatory cells (*n* = 3 for each group, magnification, 400x; Olympus BX41, Japan).

Extensively inflammatory cells in the paw tissue were observed 4 h after carrageenan injection, and the number of neutrophils increased significantly. Compared with the vehicle group, Dex (6 mg/kg), escin injection (1.8 mg/kg), and oral administration (10 mg/kg) of escin all significantly reduced the number of inflammatory cells (Figure 3).

### 3.3. Effects of Escin after Oral Administration on PGE2 and COX-2 Level in the Edema Paws

**Figure 4:**
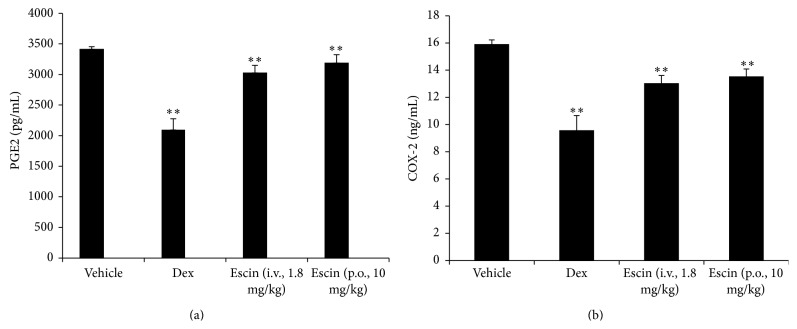
Effects of escin after oral administration on PGE2 and COX-2 level in the edema paws. Rats were assigned to four groups: vehicle group, Dex group, escin for injection group (i.v., 1.8 mg/kg), and escin for oral administration group (p.o., 10 mg/kg). *n* = 6. The significant difference was assessed using the Bonferroni post hoc test. A value of *P* < 0.05 was accepted as indicating a statistically significant difference among groups.

Compared with the vehicle group, both oral administration (10 mg/kg) and injection (1.8 mg/kg) significantly decreased the PGE2 and COX-2 levels 4 h after carrageenan injection (*P* < 0.01). There were no significant differences between them (Figure 4).

### 3.4. Effects of Escin after Oral Administration on Liver Injury of Cecal Ligation and Puncture (CLP) Induced Endotoxemic Mice

**Figure 5:**
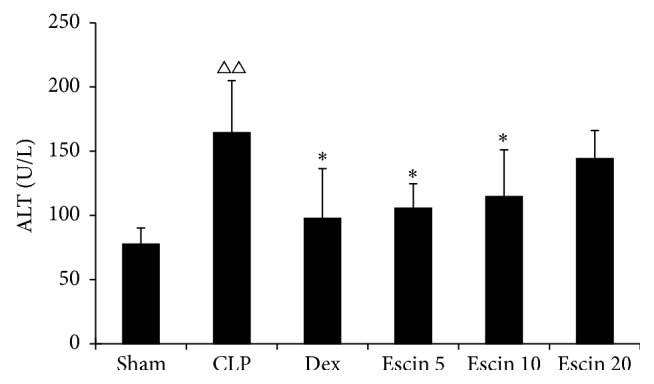
Effects of escin after oral administration on the ALT level in CLP induced endotoxemic mice. A value of *P* < 0.05 was accepted as indicating a statistically significant difference among groups. ^△△^ *P* < 0.01, compared with the sham group; ^*∗*^ *P* < 0.05, compared with the CLP group.

The level of ALT was significantly higher in the CLP group than those in the sham group (*P* < 0.01). Compared with the CLP group, the levels of ALT were remarkably lower in escin-treated groups at doses of 5 mg/kg and 10 mg/kg (*P* < 0.05), respectively (Figure 5).

**Figure 6:**
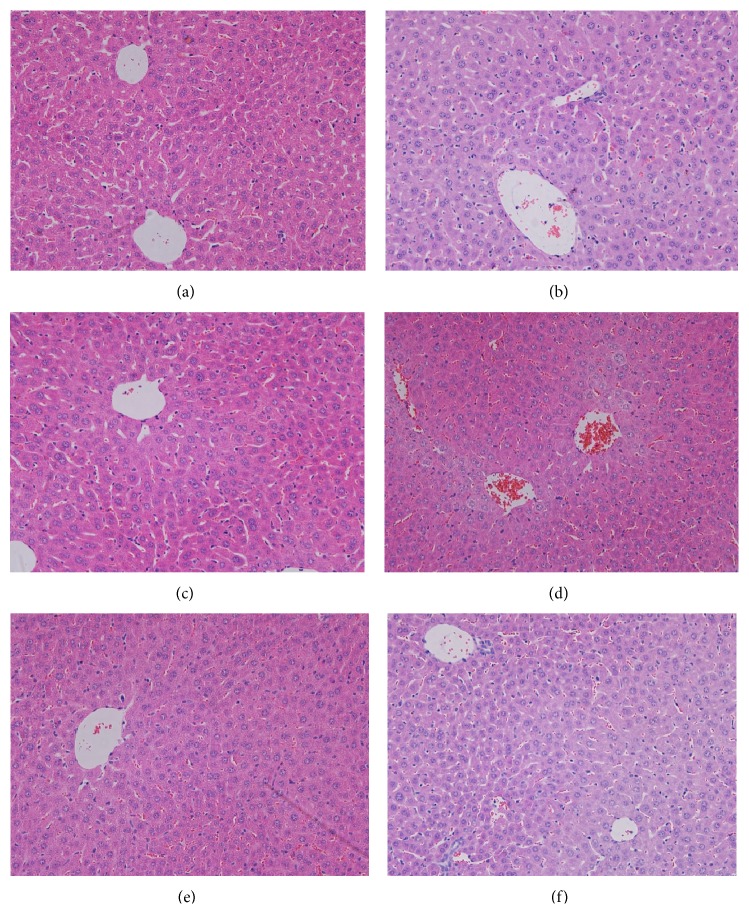
Effects of escin after oral administration on liver histological changes in CLP induced endotoxemic mice. Representative histological changes in livers obtained from different groups: (a) sham group; (b) CLP group; (c) CLP + Dex group; (d) CLP + Escin 5 mg/kg group; (e) CLP + Escin 10 mg/kg group; (f) CLP + Escin 20 mg/kg group (*n* = 3 for each group, magnification, 400x; Olympus BX41, Japan).

Liver sections from the CLP group showed significant granulocyte infiltration, disruption of cellular morphology, obvious congestion of the hepatic sinusoids, and hepatocellular necrosis compared to the sham group. Escin after oral administration (at doses of 5 mg/kg and 10 mg/kg) significantly attenuated liver damage in CLP induced endotoxemic mice (Figure 6).

### 3.5. Effects of Escin after Oral Administration on Intestinal Pathological Changes of Cecal Ligation and Puncture (CLP) Induced Endotoxemic Mice

**Figure 7:**
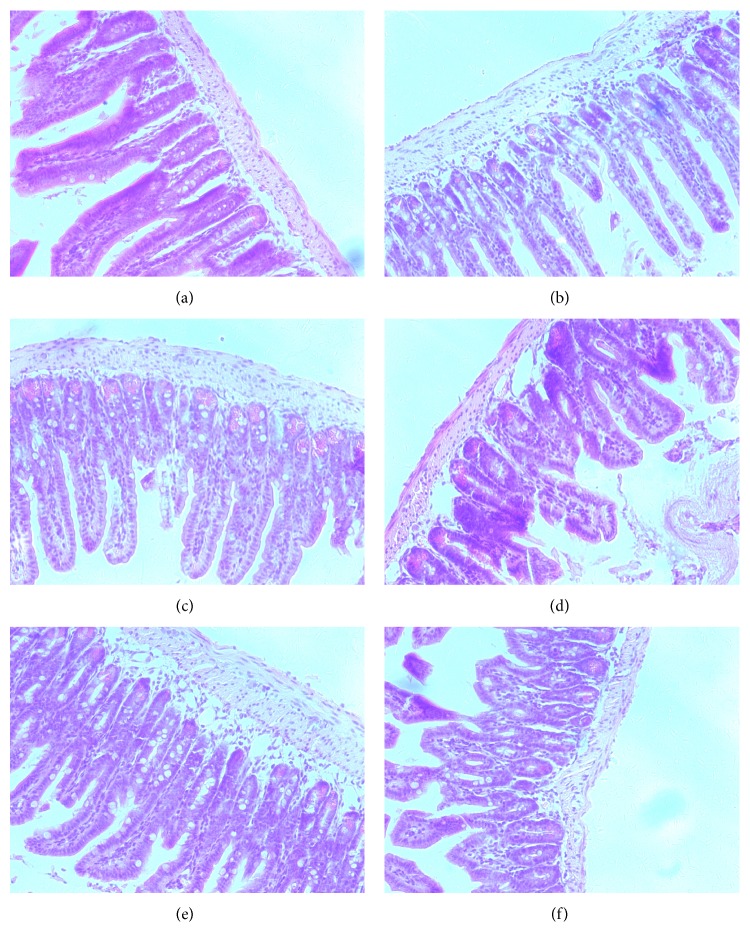
Effects of escin after oral administration on intestinal pathological changes in CLP induced endotoxemic mice. Representative histological changes in intestine obtained from different groups: (a) sham group; (b) CLP group; (c) CLP + Dex group; (d) CLP + Escin 5 mg/kg group; (e) CLP + Escin 10 mg/kg group; (f) CLP + Escin 20 mg/kg group (*n* = 3 for each group).

Compared with those of the sham control mice, the H-E stained sections of intestinal tissues from mice with CLP operation showed focal and superficial lamina propria edema and increased inflammation, epithelial layers separated from both sides of villi, and a few villi exfoliated; oral administration of escin (at doses of 5 mg/kg and 10 mg/kg) significantly attenuated damage in CLP induced endotoxemic mice (Figure 7).

### 3.6. Effects of Oral Administration of Escin on Diamine Oxidase (DAO) Activity and Endotoxin Level in Plasma

**Figure 8:**
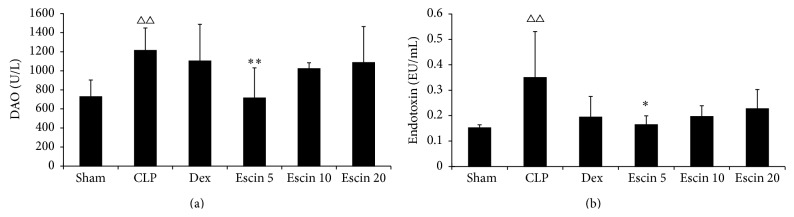
Effects of escin after oral administration on the DAO activity and endotoxin level in CLP induced endotoxemic mice. A value of *P* < 0.05 was accepted as indicating a statistically significant difference among groups. ^△△^ *P* < 0.01, compared with the sham group; ^*∗*^ *P* < 0.05 and ^*∗∗*^ *P* < 0.01, compared with the CLP group.

DAO activity and the endotoxin level in plasma were remarkably higher in the CLP group than those in the sham control group (*P* < 0.01). Compared to CLP group, levels of DAO activity and endotoxin in low dose escin-treated group were remarkably lower (*P* < 0.01, *P* < 0.05) (Figure 8).

### 3.7. Effects of Oral Administration of Escin on the Expression of Intestinal Tight Junction Protein Claudin-5

**Figure 9:**
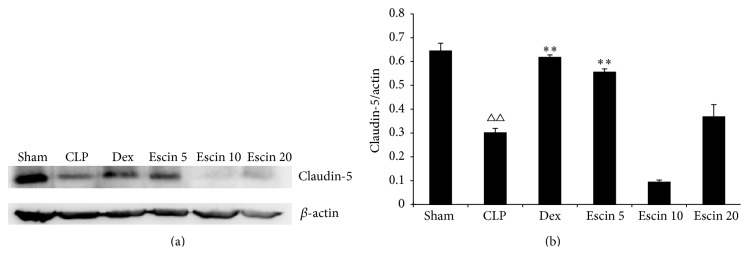
Effects of escin after oral administration on the expression of intestinal tight junction protein claudin-5. A value of *P* < 0.05 was accepted as indicating a statistically significant difference among groups. ^△△^ *P* < 0.01, compared with the sham group; ^*∗∗*^ *P* < 0.01, compared with the CLP group.

The protein expression levels of claudin-5 were significantly downregulated in CLP mice compared with those of sham mice (*P* < 0.01). Meanwhile, after low dose of escin (5 mg/kg) administration, the claudin-5 protein expression was clearly upregulated (*P* < 0.01) (Figure 9).

## 4. Discussion

The present study aimed to investigate the effects of oral administration of escin on acute inflammation and intestinal mucosal injury in animal models. The results showed that escin after oral administration could inhibit carrageenan-induced paw edema and decrease the production of PGE2 and COX-2. Escin after oral administration could also ameliorate endotoxin induced liver injury and intestinal mucosal injury in CLP model and increase the expression of tight junction protein claudin-5 in mice.

Our previous studies have proved that escin injection exerts potent anti-inflammatory effects without any immunosuppressive effects. However, some adverse reactions including phlebitis and allergic reaction directly affect its clinical use [12]. Oral administration of escin could avoid these contributing factors and prevent phlebitis and other adverse reactions which are caused by intravenous injection, so we choose escin tablet to observe whether oral administration of escin also has anti-inflammatory/antiedema effects.

Carrageenan-induced paw edema is a commonly used model for acute inflammation study [20, 21]. The acute inflammatory response induced by carrageenan injection involves two phases [22]. The early phase occurs during the first hour of exposure and is associated with the release of histamine, serotonin, and a small amount of PGs. The delayed phase after one hour is attributed to leucocyte infiltration and the continuation of PG generation. It is well established that PGs, modulators of inflammatory responses, have a major role in the inflammatory process. COX is the key enzyme that synthesizes PGs from arachidonic acid. In the present study, treatment with escin significantly inhibited the paw edema at 3, 4, 5, 6, 8, 12, and 24 h timing points after carrageenan injection indicating that escin exerted good anti-inflammatory effects. Furthermore, oral administration of escin decreased the production of PGE2 and COX-2 in the hind paws of carrageenan-treated rats. Previous studies showed that GCs can inhibit inflammatory cytokine-induced COX-2 expression [23]; our previous studies showed that escin exerts synergistic anti-inflammatory effects by enhancing the expression of glucocorticoid receptor (GR) [8, 24]. So it is indicated that the inhibition of COX-2 produced by escin may be associated with its action on GR.

Sepsis is characterized by a severe inflammatory response to infection. It has been recognized that intestinal epithelial barrier dysfunction and increased intestinal permeability after thermal injury, trauma, severe infection, and so forth may be the inciting incident that ultimately leads to sepsis. Sepsis can lead to multiple organ dysfunction syndrome (MODS), and liver is the common organ being involved, whose severity accounts for a high mortality rate [25].

The integrity of gut barrier is mainly maintained by tight junctions of intestinal mucosa, which are composed of a complex of proteins including the integral proteins such as claudins and occludin and the peripheral membrane proteins such as zonula occludens-1 (ZO-1) [26].

Claudins are a family of proteins that are important components of the tight junctions, where they establish the paracellular barrier that controls the flow of molecules in the intercellular space among the cells of an epithelium. VE-cadherin controls claudin-5 expression by preventing the nuclear accumulation of FoxO1 and *β*-catenin, which repress the claudin-5 promoter, providing a cross talk among endothelial junctional structures [27].

Previous studies suggest that escin not only protects gastric ulcer but also promotes gastrointestinal transit in animal models [17, 18]. Whether escin has the protective effects on intestinal epithelial barrier dysfunction is not clear. In the present study, we found that oral administration of low dose of escin ameliorates intestinal mucosal injury, reduces DAO activity, a marker for evaluation of the integrity of intestinal mucosa and endotoxin level in plasma, and protects against endotoxin induced liver injury. We also found that low dose of escin increases the expression levels of tight junction protein claudin-5. These results indicate that escin could ameliorate intestinal mucosal injury by protecting the intestinal barrier, which in turn protects against endotoxin induced organ injury in CLP mice. In addition, mid and high doses of escin did not show similar results produced by low dose of escin. The exact mechanisms are still unclear, but there are several possibilities which may contribute to the process. Studies have proved that GCs can augment intestinal epithelial barrier function in animal models [28, 29], so the protection of escin at low dose on intestinal mucosal injury could be associated with its synergistic effects with GCs by enhancing the expression of GR, which was reported in our previous study. Escin shows cytotoxic effects and induces intestinal epithelial apoptosis and further exacerbates the intestinal mucosal injury when higher dose of escin is used [30].

In conclusion, escin is a potent anti-inflammatory and antiedematous medicine and has been used clinically, but its side effects such as phlebitis affect its clinical use. The present study demonstrated, for the first time, that oral administration of escin shows similar anti-inflammatory and antiedematous effects as escin injection. Furthermore, we demonstrate that escin reduces intestinal mucosal injury in CLP induced endotoxemic models. These results provide experimental evidences for escin tablet to be widely used clinically.
